# Nanoscale heat evaluation performance using hybrid nanocomposites along vertical riga surface under robin boundary and transient concept

**DOI:** 10.1186/s11671-026-04835-9

**Published:** 2026-08-17

**Authors:** Ghulfam Sarfraz, Mohamed Arbi Khlifi, Heba G. Mohamed, Wasim Jamshed, Woinshet Defar Mergia, Adnan, Yasir Khan, Muhammad Nasir Bashir

**Affiliations:** 1 Department of Mathematics, Mohi-Ud-Din Islamic University, Nerian Sharif, Trarkhel, 12080 Azad Jammu and Kashmir Pakistan; 2 Department of Electrical Engineering, Faculty of Engineering, Islamic University of Madinah, 42351 Madinah, Saudi Arabia; 3 Department of Electrical Engineering, College of Engineering, Princess Nourah bint Abdulrahman University, P.O. Box 84428, 11671 Riyadh, Saudi Arabia; 4 Department of Computer Engineering, Biruni University, Topkapi, Istanbul, Turkey; 5 Department of Mathematics, Jimma University, Jimma, Ethiopia; 6 Department of Mathematics, University of Hafr Al Batin, 31991 Hafr Al Batin, Saudi Arabia; 7 Department of Mechanical Engineering, College of Engineering, King Faisal University, Al-Ahsa, 31982 Saudi Arabia

**Keywords:** Nanomaterial, Mechanical engineering, Thermal engineering, Robin condition, Numerical analysis

## Abstract

The heat transfer through Riga surface associated to Robin condition and transient effects is an interesting topic. The primary objectives of this work are to model a heat transfer problem for vertical Riga surface using hybrid nanocomposite properties, along with thermal radiation, mixed convection and unsteady effects. The problem governs the flow transformed into dimensionless form with the help of similarity transformations, and enhanced properties of nanofluids. The model novelty falls in the development of superior tetra nanofluid class and its comparison with traditional classes (ternary, hybrid and simple) by integrating the aforementioned parameters. After that, the RK numerical scheme is applied for the solution and the results plotted by varying the physical parameters (thermal radiation, Biot number, unsteady and mixed convection parameters). The findings reveal that Biot number from 0.2 to 0.8, thermal radiation parameter from 0.1 to 0.7, and nanoparticles concentration from 1 to 4% promisingly increases the temperature with maximum increase in tetra nanofluid case than ternary, hybrid and simple cases. However, strengthening the mixed convection (*δ *) from 0.01 to 0.04 and magnetic effects due to Riga surface controls the temperature significantly. The skin friction against ${\phi}_{1}$ from 1 to 4% shows variations from 1.0214 to 1.0051 (tetra), 1.1290 to 1.1179 (ternary), 1.1385 to 1.1304 (hybrid), and 1.1786 to 1.1740 for traditional nanofluid. The comparative heat transfer gradient improves for strong Biot number effects and enhances from 0.2707 to 0.440 (tetra), 0.1233 to 0.3915 (ternary), 0.1150 to 0.3671 (hybrid) and 0.1109 to 0.3536 for simple nanofluid with promising increase in tetra nanofluid. The comparative findings reveal key role of tetra nanofluid for heat transfer while the less thermal efficiency is investigated for other classes.

## Introduction

The heat transport mechanism in nanofluids remain an interesting and potential research direction. Ullah et al. [1] studied the MHD flow of Casson nanofluid using a stretching sheet in the presence of porous medium and concluded that the Casson nanofluid is much better to control the temperature than Newtonian fluid. Khan et al. [2] investigated the flow of Casson nanofluid past a porous medium with Newtonian heating. They examined that both velocity and temperature profiles reduce due to rising values of nanoparticles volume fraction. Sarkar et al. [3] analysed the impacts of melting process on hydromagnetic wedge flow involving Casson nanofluid and showed that the melting process maximize the resistance in the flow of momentum and thermal boundary layer whereas it decreases the skin friction and transfer rate of heat and mass. Khan et al. [4] investigated that the Cattaneo-Christov effects through a Riga plate and revealed that the velocity profile increases due to the influence of maximum cross viscous parameter. Also, the heat transfer enhances for strong thermal relaxation under implemented conditions.

The study of copper–water nanofluid under Cattaneo-Christov model is analysed by Alharbi et al. [5]. They concluded that the transfer of heat increases due to mixed convection in the case of suction but there was an opposite result in the case of injection. Ahmed et al. [6] used a Riga plate to investigate CCDD on nanofluid with irreversibility and motile microbes. According to results, the distribution of microorganism declined under higher Peclet number. Li et al. [7] analysed the impact of bioconvection along with CC heat flux involving stagnation point flow and discussed the entropy generation. They examined that the thermal relaxation time is a reason to decrease the temperature. Zubaidi et al. [8] examined the behaviour of heat and mass transfer in the existence of Riga surface and examined that the porous medium is responsible to reduce the boundary layer. Recent investigations [9, 10] shown considerable attention of the researchers [11, 12] for nanofluids study. Nadeem et al. [13] worked on 3D heat transfer using a Riga plate. The flow is induced due to stretching surface with variable thermal conductivity.

The use of Riga surface for heat transfer subject to the heat source and sink involvement is studied by Akolade et al. [14]. They noticed that reduction of the boundary layer is dominant for Williamson number but heat source prevailed the temperature in the case of enhancing nanoparticles concentration. Khan et al. [15] studied the bio-convection nanofluid flow of Eyring-Powell fluid in the presence of CC model. They examined thermal progress of the model developed due to the phenomenon of heat source/sink. Recent attempts for nanofluids applications for heat transfer in different configurations have been reported in the Refs. [16–18], and [19]. These studies performed for different fluids under semi infinite conditions and obtained remarkable heat transfer results. Jayanthi and Niranjan [20] analysed the impacts of Joule heating and viscous dissipation on the heat transfer using a vertical surface. According to results, the increase in temperature and concentration occur due to rising values of Schmidt number.

Saleem and Tufail [21] studied the Casson nanofluid flow in the presence of bio-convection based and concluded that the Levis number is source of increasing the microorganism mobility. Khan et al. [22] numerically investigated the nanofluid flow in bioconvection using a porous medium. They found that the nanoparticles concentration field reduces due to Schmidt number. Also, the gyrotactic microorganism concentration is increased because of higher Peclet number. Ahmed et al. [23] studied the influence of mixed convection hydromagnetic peristaltic flow in the presence of Joule heating in curved channel. The results explored the fact that the maximum values of thermal Grashof number and Hartmann number are reason of increasing the temperature profile. On the other hand, higher values of thermal Grashof number and Hartmann number are responsible for reduced mass transfer. Jamalabadi et al. [24] investigated the impacts of Joule heating, viscous dissipation and thermal radiation on the flow problem. They revealed that the temperature has minimal variations under higher magnetic field. Furthermore, they observed that higher thermal performance of the model when thermal radiation effects increases. But there was opposite result for thermal boundary layer and the Nusselt number at the wall. The studies of nanofluids efficiency using distinct primary fluids and variety of nanoparticles under certain operating conditions have been discussed in the Refs. [25–28], and [29].

Although, the heat transfer through Riga surface has received substational interest of the researchers by focusing on traditional nanofluids (ternary, mono and hybrid types). However, the study for tetra nanofluid is remains an open window to discuss their effectiveness in comparison with previous classes under combined influence of mixed convection, convective heating, thermal radiation and thermal slip effects and unsteady flow on the heat performance of tetra nanofluid through vertically configured Riga plate. This framework is remains unexplored in single mathematical model. These physical parameters strongly interact and potentially influenced the thermal and momentum transport, overlooking their combined contribution leads to an inadequate understanding of the fundamental heat transfer mechanism. Thus, the present study investigates a detailed numerical analysis of tetra nanofluid dynamics through vertical Riga plate by including all these parameters in unified problem formulation. The contribution of this research falls in providing a detailed comparative heat transport investigation under mentioned parameters which will highlight the valuable role of four nanoparticles mixture.

## Model development

This work presented the tetra nanofluid flow of time dependent model over a stretchy vertical Riga sheet. The thermal slip and heat convective condition are taken for thermal enhancement. Furthermore, Lorentz forces also called controlling forces generated through the sequence of magnets.

The current model is presented through the following mathematical expressions [30]:

1 $$\frac{\partial u}{\partial x}+\frac{\partial v}{\partial y}=0$$

2 $$\frac{\partial u}{\partial t}+u\frac{\partial u}{\partial x}+v\frac{\partial u}{\partial y}=\frac{\partial {u}_{\infty }}{\partial t}+\frac{{u}_{\infty }\partial {u}_{\infty }}{\partial x}+\frac{{\mu}_{tet}}{{\rho}_{tet}}\frac{{\partial }^{2}u}{\partial {y}^{2}}+\frac{{M}_{^\circ }{\sigma}_{tet}}{8\pi {\rho}_{tet}}{e}^{\left(\frac{-\pi y}{a}\right)}$$

3 $$\frac{\partial T}{\partial t}+u\frac{\partial T}{\partial x}+v\frac{\partial T}{\partial y}=\frac{{k}_{tet}}{{\left(\rho {c}_{p}\right)}_{tet}}\frac{{\partial }^{2}T}{\partial {y}^{2}}+\frac{16\sigma {T}^{3}}{3{k}^{*}{\left(\rho {c}_{p}\right)}_{tet}}\frac{{\partial }^{2}T}{\partial {y}^{2}}$$

With

4 $$v=0,u=0, {h}_{f}\frac{\partial T}{\partial y}={h}_{f}\left({T}_{f}-T\right)+L\frac{{k}_{tet}}{h}\frac{\partial T}{\partial y}$$

5 $$u={u}_{e}, T={T}_{\infty } as y\to \infty $$

Taking the applicable transformation as:

6 $$ \beta = \frac{{T - T_{\infty } }}{{T_{f} - T_{\infty } }}, \eta = \sqrt {\frac{a}{{v_{f} \left( {1 - \alpha t} \right)}}} y, u = \frac{ax}{{\left( {1 - \alpha t} \right)}}F^{\prime}, v = - \sqrt {\frac{{a\nu_{f} }}{{\left( {1 - \alpha t} \right)}}} F $$

The updated characteristics [31] (Table 1) of nanofluid using Tetra model are characterized in the subsequent table. Further, the individual properties [32] of nanofluid mixture are enlisted in Table 1. These NPs uniformly mixed in the primary fluid to make the tetra nanofluid.

**Table 1:** Tetra nanofluid properties

S.no	Models
1	${\widehat{\mu }}_{tet}={\widehat{\mu }}_{f}{\left[{\left(1-{\widehat{\phi }}_{1}\right)}^{2.5}{\left(1-{\widehat{\phi }}_{2}\right)}^{2.5}{\left(1-{\widehat{\phi }}_{3}\right)}^{2.5}{\left(1-{\widehat{\phi }}_{4}\right)}^{2.5}\right]}^{-1}$
2	${\rho}_{tet}=\left[\left(1-{\widehat{\phi }}_{4}\right)\left(\left(1-{\widehat{\phi }}_{3}\right)\left(\left(1-{\widehat{\phi }}_{2}\right)\left(\left(1-{\widehat{\phi }}_{1}\right)+\frac{{\widehat{\phi }}_{1}{\rho}_{s1}}{{\rho}_{f}}\right)+\frac{{\widehat{\phi }}_{2}{\rho}_{s2}}{{\rho}_{f}}\right)+\frac{{\widehat{\phi }}_{3}{\rho}_{s3}}{{\rho}_{f}}\right)+\frac{{\widehat{\phi }}_{4}{\rho}_{s4}}{{\rho}_{f}}\right]$
3	${(\rho {c}_{p})}_{tet}=\left[\left(1-{\widehat{\phi }}_{4}\right)\left(\left(1-{\widehat{\phi }}_{3}\right)\left(\left(1-{\widehat{\phi }}_{2}\right)\left(\left(1-{\widehat{\phi }}_{1}\right)+\frac{{\widehat{\phi }}_{1}{(\rho {c}_{p})}_{s1}}{{(\rho {c}_{p})}_{f}}\right)+\frac{{\widehat{\phi }}_{2}{(\rho {c}_{p})}_{s2}}{{(\rho {c}_{p})}_{f}}\right)+\frac{{\widehat{\phi }}_{3}{(\rho {c}_{p})}_{s3}}{{(\rho {c}_{p})}_{f}}\right)+\frac{{\widehat{\phi }}_{4}{(\rho {c}_{p})}_{s4}}{{(\rho {c}_{p})}_{f}}\right]$
4	${(\rho \beta )}_{tet}=\left[\left(1-{\widehat{\phi }}_{4}\right)\left(\left(1-{\widehat{\phi }}_{3}\right)\left(\left(1-{\widehat{\phi }}_{2}\right)\left(\left(1-{\widehat{\phi }}_{1}\right)+\frac{{\widehat{\phi }}_{1}{(\rho \beta )}_{s1}}{{(\rho \beta )}_{f}}\right)+\frac{{\widehat{\phi }}_{2}{(\rho \beta )}_{s2}}{{(\rho \beta )}_{f}}\right)+\frac{{\widehat{\phi }}_{3}{(\rho \beta )}_{s3}}{{(\rho \beta )}_{f}}\right)+\frac{{\widehat{\phi }}_{4}{(\rho \beta )}_{s4}}{{(\rho \beta )}_{f}}\right]$
5	$\frac{{k}_{tet}}{{k}_{f}}=\frac{\left({k}_{\mathrm{s}4}+2{k}_{ter}-2{\widehat{\phi }}_{4}\left({k}_{ter}-{k}_{\mathrm{s}4}\right)\right)}{({k}_{\mathrm{s}4}+2{k}_{ter}+{\widehat{\phi }}_{4}({k}_{ter}-{k}_{\mathrm{s}4})}$ $\frac{{k}_{ter}}{{k}_{f}}=\frac{\left({k}_{\mathrm{s}3}+2{k}_{hyb}-2{\widehat{\phi }}_{3}\left({k}_{hyb}-{k}_{\mathrm{s}3}\right)\right)}{({k}_{\mathrm{s}3}+2{k}_{hyb}+{\widehat{\phi }}_{3}({k}_{hyb}-{k}_{\mathrm{s}3})}$ $\frac{{k}_{hyb}}{{k}_{f}}=\frac{\left({k}_{\mathrm{s}2}+2{k}_{nano}-2{\widehat{\phi }}_{2}\left({k}_{nano}-{k}_{\mathrm{s}2}\right)\right)}{({k}_{\mathrm{s}2}+2{k}_{nano}+{\widehat{\phi }}_{2}({k}_{nano}-{k}_{\mathrm{s}2})}$ $\frac{{k}_{nano}}{{k}_{f}}=\frac{({k}_{\mathrm{s}1}+2{k}_{f}-2{\widehat{\phi }}_{1}({k}_{f}-{k}_{\mathrm{s}1}))}{({k}_{\mathrm{s}1}+2{k}_{f}+{\widehat{\phi }}_{1}({k}_{f}-{k}_{\mathrm{s}1})}$
6	$\frac{{\sigma}_{tet}}{{\sigma}_{f} }=\frac{({\sigma}_{s4}+2{\sigma}_{ter}-2{{\widehat{\phi }}_{4}(\sigma }_{ter}-{\sigma}_{s4}))}{{\sigma}_{s4}+2{\sigma}_{ter}+{\widehat{\phi }}_{4}\left({\sigma}_{ter}-{\sigma}_{s4}\right)}$ $\frac{{\sigma}_{ter}}{{\sigma}_{f} }=\frac{({\sigma}_{s3}+2{\sigma}_{hyb}-2{{\widehat{\phi }}_{3}(\sigma }_{hyb}-{\sigma}_{s3}))}{{\sigma}_{s3}+2{\sigma}_{hyb}+{\widehat{\phi }}_{3}\left({\sigma}_{hyb}-{\sigma}_{s3}\right)}$ $\frac{{\sigma}_{hyb}}{{\sigma}_{f} }=\frac{({\sigma}_{s2}+2{\sigma}_{nano}-2{{\widehat{\phi }}_{2}(\sigma }_{nano}-{\sigma}_{s2}))}{{\sigma}_{s2}+2{\sigma}_{nano}+{\widehat{\phi }}_{2}\left({\sigma}_{nano}-{\sigma}_{s2}\right)}$ $\frac{{\sigma}_{nano}}{{\sigma}_{f} }=\frac{({\sigma}_{s1}+2{\sigma}_{f}-2{{\widehat{\phi }}_{1}(\sigma }_{f}-{\sigma}_{s1}))}{{\sigma}_{s1}+2{\sigma}_{f}+{\widehat{\phi }}_{1}({\sigma}_{f}-{\sigma}_{s1})}$

Components	$\rho (kg/{m}^{3})$	*σ (S/m)*	${C}_{p} (J/kgK)$	*k (W/mK)*
CuO	*6320*	$2.7\times {10}^{-8}$	*531.8*	*76.5*
Ag	*10500*	$6.3\times {10}^{7}$	*235*	*429*
Fe_3_O_4_	*5200*	*25000*	*670*	*6.0*
Au	*19282*	$4.1\times {10}^{-6}$	*129*	*310*
MoS_2_	*5060*	$2.09\times {10}^{-5}$	*397.21*	*904.4*
H_2_O	*997.1*	*0.05*	*4179*	*0.613*

The usage of above transformation reduced the present model in following form:

7 $$ \frac{{\mu_{{tet/\mu_{f} }} }}{{\rho_{tet} /\rho_{f} }}F^{\prime\prime\prime} + 1 + H_{a} \frac{{e^{{ - \gamma_{1} \eta }} }}{{\rho_{tet} /\rho_{f} }} + FF^{\prime\prime} - F^{{\prime^{2} }} - \frac{{\gamma_{2} }}{{\frac{{\rho_{tet} }}{{\rho_{f} }}}}\left[ {\frac{\eta }{2}F^{\prime\prime} + F^{\prime} - 1} \right] + \left( {\rho \beta^{*} } \right)_{tet} /\left( {\rho \beta^{*} } \right)_{f} \delta \beta = 0 $$

8 $$ \frac{{k_{tet} /k_{f} }}{{\left( {\rho C_{p} } \right)_{tet} /\left( {\rho C_{p} } \right)_{f} }}\left( {1 + \frac{4}{3}Rd} \right)\beta^{\prime\prime} + \Pr \left[ {F\beta^{\prime} + \frac{{\gamma_{2} }}{{2\left( {\rho C_{p} } \right)_{tet} /\left( {\rho C_{p} } \right)_{f} }}\eta \beta^{\prime}} \right] = 0 $$

with the conditions that:

9 $$ F\left( 0 \right) = 0, F^{\prime}\left( 0 \right) = \gamma_{3} , F^{\prime}\left( \infty \right) = 1, \beta \left( \infty \right) = 0 $$

and

10 $$ \beta^{\prime}\left( 0 \right) = \frac{{ - B_{i} \left( {1 - \beta \left( 0 \right)} \right)}}{{\left( {1 + \gamma_{4} } \right)}} $$

The physical quantities in Eqs. (7–8) are unsteady parameter ${\gamma}_{2}=\alpha /a$, thermal radiation $Rd=\frac{16{\sigma }^{*}{T}_{\infty }^{3}}{3{k}^{*}{k}_{f}}$, Prandtl number $Pr={\nu}_{f}/{\alpha}_{f}$, Grashoff number $\delta =G{r}_{x}/R{e}_{x}^{2}$, Hartmann number ${H}_{a}=\frac{{J}_{o}{M}_{o}\pi }{{u}_{w}^{2}{\rho}_{f}8}$, stretching number ${\gamma}_{3}={u}_{e}/{u}_{w}$, thermal slip ${\gamma}_{4}=L{\left(\frac{a}{{\nu}_{f}\left(1-\alpha t\right)}\right)}^{1/2}$ and Biot number ${B}_{i}=\frac{{h}_{f}}{{k}_{f}}\sqrt{{\nu}_{f}(1-\alpha t)/a}$. Further, the engineering quantities are expressed as below.

$$ C_{fx} = \frac{{\mu_{tet} }}{{\rho_{f} u_{w}^{2} }}\left( {\frac{\partial \mathop u\limits }{{\partial z}}} \right)_{z = 0} ,C_{fy} = \frac{{\mu_{tet} }}{{\rho_{f} u_{w}^{2} }}\left( {\frac{\partial \mathop v\limits }{{\partial z}}} \right)_{z = 0} ,Nu_{x} = - \frac{{k_{tet} }}{{k_{f} \left( {T_{f} - T_{\infty } } \right)}}\left( {\frac{\partial T}{{\partial z}}} \right)_{z = 0} + x\left( {q_{r} } \right)_{z} $$

The nondimensionalized forms of manufacturing factors are:

11 $$ C_{fx} Re_{x}^{{{\raise0.7ex\hbox{$1$} \!\mathord{\left/ {\vphantom {1 2}}\right.\kern-0pt} \!\lower0.7ex\hbox{$2$}}}} = \left[ {\frac{{\mu_{tet} }}{{\mu_{f} }}} \right]F^{\prime\prime}\left( 0 \right), C_{fy} Re_{x}^{{{\raise0.7ex\hbox{$1$} \!\mathord{\left/ {\vphantom {1 2}}\right.\kern-0pt} \!\lower0.7ex\hbox{$2$}}}} = \left[ {\frac{{\mu_{tet} }}{{\mu_{f} }}} \right]G^{\prime\prime}\left( 0 \right) $$

12 $$ Nu_{x} Re_{x}^{{ - {\raise0.7ex\hbox{$1$} \!\mathord{\left/ {\vphantom {1 2}}\right.\kern-0pt} \!\lower0.7ex\hbox{$2$}}}} = - \left( {\frac{{k_{tet} }}{{k_{f} }} + \frac{4}{3}Rd} \right)\beta^{\prime}\left( 0 \right), $$

$Re=\frac{ux}{\nu f}$ denoted the local Reynolds number.

## Mathematical analysis

The reduced Eqs. (7–10) provides a two point BVP [33, 34] because the conditions are given at the surface and ambient position. Thus, the first order system is the appropriate form for implementation of the scheme and then treated through RK scheme [35, 36], coupling with shooting method [37, 38]. The *∞ * condition is restricted to appropriate large *∞ * value i.e. $\eta ={\eta}_{\infty }$, where the ambient condition properly satisfied. For this, introducing the following transformations.

13 $${\mathcal{J}}_{1}=F,{\mathcal{J}}_{2}={F}^{\prime},{\mathcal{J}}_{3}={F}^{{\prime}{\prime}},{\mathcal{J}}_{3}^{\prime}={F}^{{\prime}{\prime}{\prime}},{\mathcal{J}}_{4}=\beta ,{\mathcal{J}}_{5}={\beta }^{\prime},{\mathcal{J}}_{5}^{\prime}={\beta }^{{\prime}{\prime}},$$

Accordingly, the model equations converted in the following form.

14 $${\mathcal{J}}_{1}^{\prime}={\mathcal{J}}_{2},{\mathcal{J}}_{2}^{\prime}={\mathcal{J}}_{3},$$

15 $$\frac{{\mu}_{mthnf}}{{\rho}_{mthnf}}{\mathcal{J}}_{3}^{\prime}=-\left[1+{H}_{a}\frac{{e}^{-{\gamma}_{1}\eta }}{{\rho}_{mthnf}}+{\mathcal{J}}_{1}{\mathcal{J}}_{3}-{{\mathcal{J}}_{2}}^{2}-\frac{{\gamma}_{2}}{{\rho}_{mthnf}}\left[\frac{\eta }{2}{\mathcal{J}}_{3}+{\mathcal{J}}_{2}-1\right]+{\left(\rho \beta \right)}_{mthnf}\delta {\mathcal{J}}_{4}\right],$$

16 $${\mathcal{J}}_{4}^{\prime}={\mathcal{J}}_{5},$$

17 $$\frac{{k}_{mthnf}}{{\left(\rho {C}_{p}\right)}_{mthnf}}\left(1+\frac{4}{3}Rd\right){\mathcal{J}}_{5}^{\prime}=\mathit{Pr}\left[{\mathcal{J}}_{1}{\mathcal{J}}_{5}+\frac{{\gamma}_{2}}{2{\left(\rho {C}_{p}\right)}_{mthnf}}\eta {\mathcal{J}}_{1}\right],$$

The BCs associated to the model are organized in the following way.

18 $${\mathcal{J}}_{1}\left(0\right)=0,{\mathcal{J}}_{2}\left(0\right)={\gamma}_{3},{\mathcal{J}}_{2}\left(\infty \right)=1,{\mathcal{J}}_{4}\left(\infty \right)=0,{\mathcal{J}}_{5}\left(0\right)=-\frac{{B}_{i}\left(1-{\mathcal{J}}_{4}\left(0\right)\right)}{\left(1+{\gamma}_{4}\right)},$$

The unknown ${\mathcal{J}}_{3}(0)$ and ${\mathcal{J}}_{4}\left(0\right)$ are assigned as shooting parameters and defined as below.

19 $${\mathcal{J}}_{3}\left(0\right)={S}_{1}, {\mathcal{J}}_{4}\left(0\right)={S}_{2},$$

Further, the ICs implemented for RK4 scheme are as under.

20 $${\mathcal{J}}_{1}\left(0\right)=0,{\mathcal{J}}_{2}\left(0\right)={\gamma}_{3},{\mathcal{J}}_{3}\left(0\right)={S}_{1},{\mathcal{J}}_{4}\left(0\right)={S}_{2},{\mathcal{J}}_{5}\left(0\right)=-\frac{{B}_{i}\left(1-{S}_{2}\right)}{\left(1+{\gamma}_{4}\right)},$$

For arranged values of the parameters ${S}_{1}$ and ${S}_{2}$, the system is then integrated from *0* to ${\eta}_{\infty }$ with the help of RK4 scheme and the solution will be accepted upon satisfaction of the terminal BCs.

21 $${R}_{1}={\mathcal{J}}_{2}\left(\infty \right)-1=0,{R}_{2}={\mathcal{J}}_{4}\left(\infty \right)=0,$$

The shooting constraints are updated iteratively until the following convergence obtained.

22 $$\left|{R}_{1}\right|\le {10}^{-6},\left|{R}_{2}\right|\le {10}^{-6},$$

The RK4 scheme adopted using the following formulas and then achieved the final results.

23 $${\mathcal{J}}_{(i+1)}={\mathcal{J}}_{i}+\frac{h}{6}\left[{\mathcal{K}}_{1}+2{\mathcal{K}}_{2}+2{\mathcal{K}}_{3}+{\mathcal{K}}_{4}\right],$$

24 $${\mathcal{K}}_{1}=g\left({\eta}_{i},{\mathcal{J}}_{i}\right),$$

25 $${\mathcal{K}}_{2}=g\left({\eta}_{i}+\frac{h}{2},{\mathcal{J}}_{i}+\frac{h{\mathcal{K}}_{1}}{2}\right),$$

26 $${\mathcal{K}}_{3}=g\left({\eta}_{i}+\frac{h}{2},{\mathcal{J}}_{i}+\frac{h{\mathcal{K}}_{2}}{2}\right),$$

27 $${\mathcal{K}}_{4}=g\left({\eta}_{i}+h,{\mathcal{J}}_{i}+h{\mathcal{K}}_{3}\right),$$

28 $$\mathcal{J}={\left[{\mathcal{J}}_{1},{\mathcal{J}}_{2},{\mathcal{J}}_{3},{\mathcal{J}}_{4}.{\mathcal{J}}_{5}\right]}^{t},$$

After this, the iterative procedure sustained until the desired convergence obtained and then the convergent solutions used to the velocity, temperature, skin friction and Nusselt number computations.

## Results and discussion

This section provides a detailed investigation of tetra nanofluid (composition of four types of nanoparticles and fluid), ternary (three types of nanoparticles saturated in the base fluid), hybrid and simple nanofluids which are two and single nanoparticles saturation in the base fluids, respectively.

**Fig. 1:**
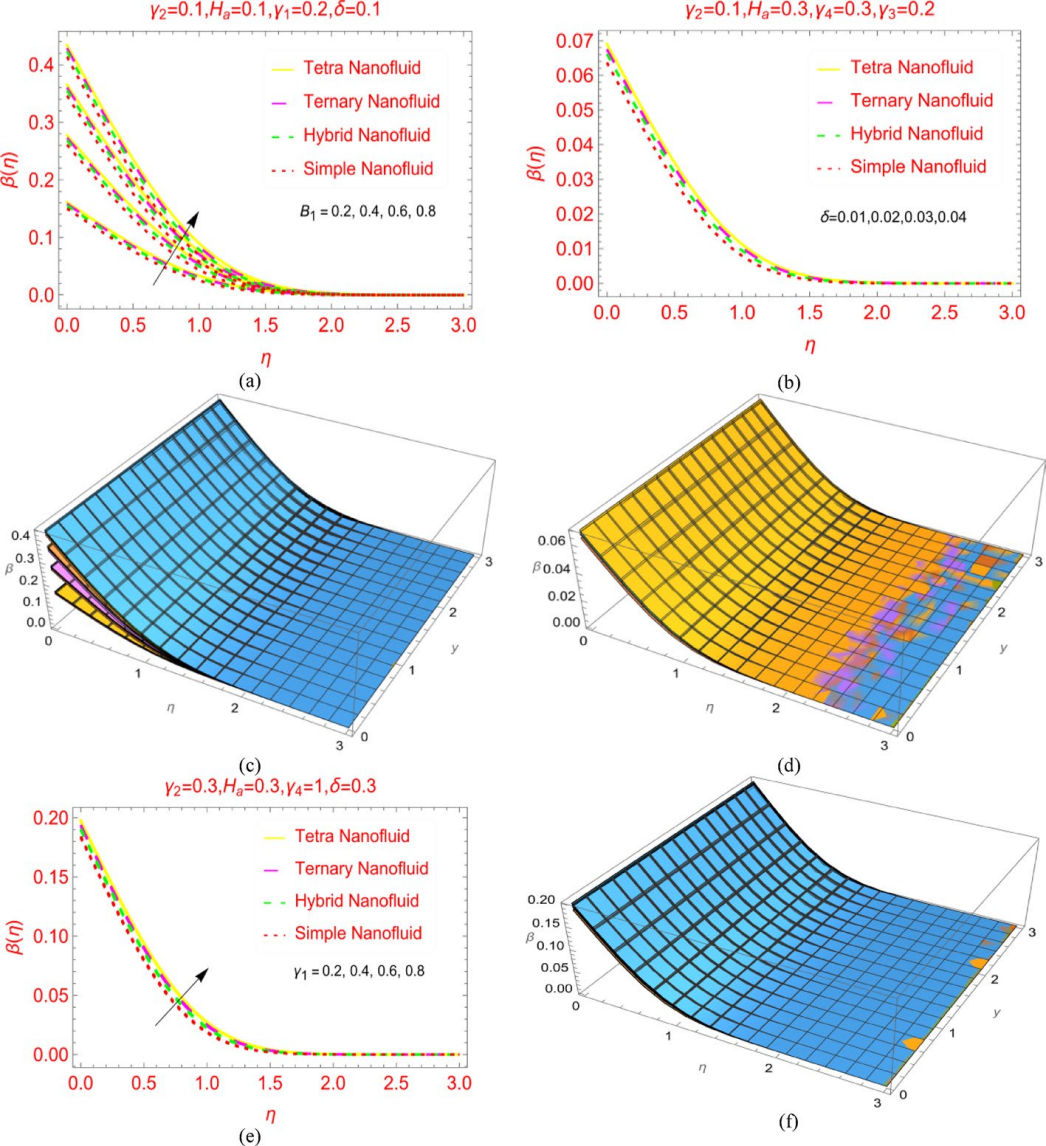
The temperature variations under increasing values of Biot number ${B}_{1}$ and mixed convection *δ * (a–d), and ${\gamma}_{1}$ in (e–f)

Figure 1a–f highlighting the role of Biot (${B}_{1}$), mixed convection (*δ *) and ${\gamma}_{1}$ on the comparative temperature of nanofluids. Figure 1a represents significant increase for all nanofluids under higher Biot number, while more pronounced thermal behaviour is noticed for tetra nanofluid than other suspensions. Physically, the ${B}_{1}$ is the ratio of convective type heat at the surface to conductive heat transport inside the nanofluid. The higher ${B}_{1}$ enhances the heat exchanges between heated Riga sheet and the adjacent nanofluid and injecting extra thermal energy into the BL (boundary layer). This injecting energy disperses farther from the surface, providing a thicker thermal boundary layer and enhanced nanofluids temperature. As, the tetra nanofluid is suspension of four types of NPs and its heat capacity and thermal conductivity are considerably higher than simple, hybrid and ternary nanofluids. Thus, it transfers thermal energy efficiently which results higher temperature. Figure 1b–d reflect the influence of mixed convection (*δ *) which causes a minimal variation in the temperature. This weak influence is due to buoyancy force which modifies the momentum transport while thermal diffusion remains mainly governed by convection and conduction under implemented conditions. Similarly, Fig. 1e–f highlights the marginal variations in the temperature which indicates the weak contribution of ${\gamma}_{1}$. From all these results, the tetra nanofluid provides dominant role in the temperature than other classes of nanofluids.

**Fig. 2:**
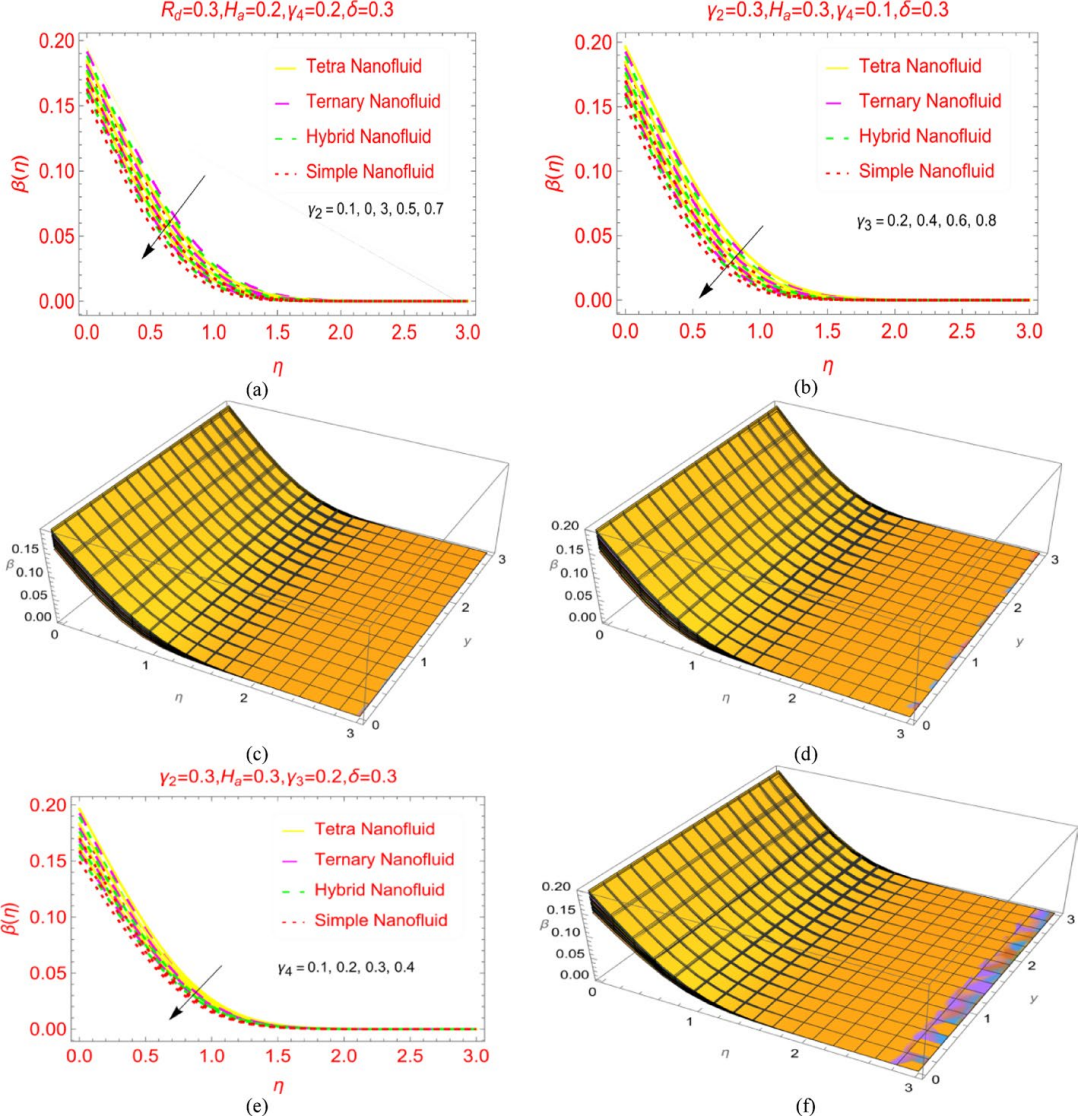
The temperature variations under varying unsteady ${\gamma}_{2}$, and stretching parameter ${\gamma}_{3}$ in (a-d) and thermal slip parameter ${\gamma}_{4}$ in (e–f)

Figure 2a–f illustrates the impact of unsteady ${\gamma}_{2}$, stretching ${\gamma}_{3}$ and thermal slip ${\gamma}_{4}$ parameters on $\beta (\eta )$. Figure 2a indicates that enlarging the unsteady parameter produces a significant reduction in the temperature which rapid decline in simple nanofluid. Physically, higher unsteady phenomena accelerate the temporal development of the BL and diminishes the duration during which the nanofluid particles remain exposed near the heated surface. The short time suppresses conductive diffusion process of the heat in the nanofluid from the surface and reduces TBL thickness and dropping the temperature. The tetra nanofluid has improved thermal conductivity and heating storge, thus it has relatively improved thermal energy despite the higher unsteadiness. However, the simple nanofluid provides excellent cooling. Figure 2b–d reveals diminishing behaviour of the temperature w.r.t stretching surface ${\gamma}_{3}$. Physically, the higher stretching of the surface accelerates the nanofluids adjacent to the Riga sheet, enhancing the convective heat transfer and replacing the heat fluid particles with cooler ambient nanofluid. This enhanced renewal of the nanofluids controls thermal accumulation near the Riga surface and suppresses the thermal progress. The thermal slip in Fig. 2e highlighting the thermal slip interaction which reduces the temperature at the surface and nanofluid interface. Thus, it decreases the amount of heat transfer from surface to the nanofluids. In this result, thermal boundary layer becomes thinner and declines the temperature. Further, the 3D (Fig. 2c, d, and f) results also supporting these trends by unveiling gradually thinner TBL with higher impact of ${\gamma}_{2}, {\gamma}_{3}$ and ${\gamma}_{4}$. However, the tetra nanofluid shows dominant role in heat distribution.

**Fig. 3:**
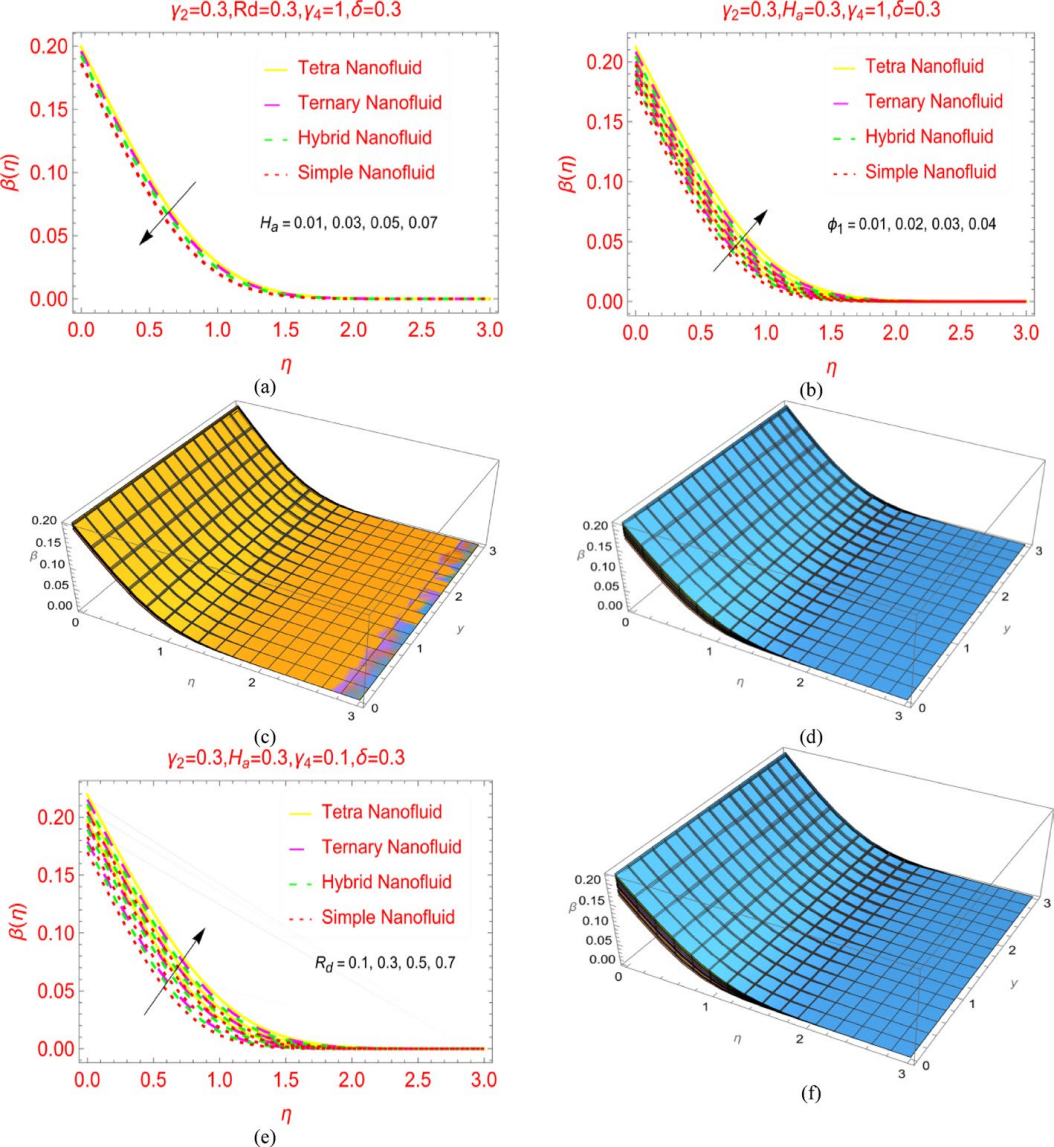
The temperature variations under varying modified Hartmann number ${H}_{a}$ and nanoparticles concentration ${\phi}_{1}$ in (a–d) and thermal radiation *Rd* in (e–f)

The response of temperature distribution due to ${H}_{a}$, ${\phi}_{1}$ and *Rd* is furnished in Fig. 3a–f, respectively. Figure 3a–c exhibit that higher ${H}_{a}$ slowly diminishes the temperature. Physically, the strong magnetic field due to fixed magnets in the geometry generates Lorentz force that weaken the convective heat mechanism. Figure 3b–d elucidating considerable increase due to concentration ${\phi}_{1}$ which elevates thermal conductivity and directly enhances the temperature in boundary layer. The nanofluids properties (thermal diffusivity, heat capacity and thermal and electrical conductivities) become enhance; thus, the temperature improves significantly along the Riga surface. Enhanced performed is examined for tetra nanofluid due to influence of four sort of NPs and their thermophysical properties. Figure 3e–f depict that *Rd* has great influence in the temperature field variations. These playing the role of additional source by providing radiative energy to the nanofluids and enhances internal energy which boosts the temperature. The 3D variations also confirm that higher ${\phi}_{1}$ and *Rd* favors the temperature while ${H}_{a}$ provides minor variations. The tetra nanofluid achieves higher temperature distribution then other classes.

Table 2 presented the Skin friction variations in response of increasing values of various physical parameters for the cases of tetra, ternary, hybrid and simple nanofluids. The higher concentration is reason to reduces the skin friction. The skin friction decreases against the rising ${\gamma}_{3}$ and ${\gamma}_{1}$ in all cases of the fluids. The tetra, ternary, hybrid and nanofluids observed enhancing scenario because of increasing ${H}_{a}$, ${\gamma}_{2}$, ${R}_{d}$, ${B}_{1}$ and *δ *. But the opposite trends are noticeable due to strong thermal slip effects ${\gamma}_{4}$.

**Table 2:** Skin Friction variations under varying the parameters

Ranges of the parameters									Skin friction			
									Tetra	Ternary	Hybrid	Nano
${\upphi}_{1}$	${\gamma}_{3}$	${\gamma}_{1}$	${H}_{a}$	${\gamma}_{2}$	${R}_{d}$	${B}_{1}$	*δ *	${\gamma}_{4}$				
*0.01*	*0.1*	*0.1*	*0.1*	*0.1*	*0.1*	*0.1*	*0.1*	*0.1*	*1.0214*	*1.1290*	*1.1385*	*1.1786*
*0.02*	–	–	–	–	–	–	–	–	*1.0162*	*1.1256*	*1.1362*	*1.1775*
*0.03*	–	–	–	–	–	–	–	–	*1.0107*	*1.1219*	*1.1335*	*1.1759*
*0.04*	–	–	–	–	–	–	–	–	*1.0051*	*1.1179*	*1.1304*	*1.1740*
–	*0.1*	–	–	–	–	–	–	–	*1.1138*	*1.2311*	*1.2415*	*1.2853*
–	*0.2*	–	–	–	–	–	–	–	*1.0214*	*1.1290*	*1.1385*	*1.1786*
–	*0.3*	–	–	–	–	–	–	–	*0.9215*	*1.0189*	*1.0276*	*1.0639*
–	*0.4*	–	–	–	–	–	–	–	*0.8145*	*0.9011*	*0.9091*	*0.9415*
–	–	*0.1*	–	–	–	–	–	–	*1.1162*	*1.2346*	*1.2452*	*1.2895*
–	–	*0.2*	–	–	–	–	–	–	*1.1138*	*1.2311*	*1.2415*	*1.2853*
–	–	*0.3*	–	–	–	–	–	–	*1.1116*	*1.2280*	*1.2382*	*1.2815*
–	–	*0.4*	–	–	–	–	–	–	*1.1096*	*1.2253*	*1.2352*	*1.2782*
–	–	–	*0.1*	–	–	–	–	–	*1.1162*	*1.2346*	*1.2452*	*1.2895*
–	–	–	*0.2*	–	–	–	–	–	*1.1576*	*1.2912*	*1.3062*	*1.3579*
–	–	–	*0.3*	–	–	–	–	–	*0.3357*	*1.3473*	*1.3666*	*1.4255*
–	–	–	*0.4*	–	–	–	–	–	*0.3787*	*1.4030*	*1.4265*	*1.4926*
–	–	–	–	*0.1*	–	–	–	–	*1.0912*	*1.1996*	*1.2075*	*1.2469*
–	–	–	–	*0.2*	–	–	–	–	*1.1037*	*1.2172*	*1.2264*	*1.2683*
–	–	–	–	*0.3*	–	–	–	–	*1.1162*	*1.2346*	*1.2452*	*1.2895*
–	–	–	–	*0.4*	–	–	–	–	*1.1286*	*1.2519*	*1.2638*	*1.3105*
–	–	–	–	–	*0.1*	–	–	–	*1.0912*	*1.1996*	*1.2075*	*1.2469*
–	–	–	–	–	*0.2*	–	–	–	*1.0917*	*1.2001*	*1.2080*	*1.2474*
–	–	–	–	–	*0.3*	–	–	–	*1.0921*	*1.2005*	*1.2084*	*1.2478*
–	–	–	–	–	*0.4*	–	–	–	*1.0925*	*1.2009*	*1.2088*	*1.2482*
–	–	–	–	–	–	*0.1*	–	–	*1.0912*	*1.1996*	*1.2075*	*1.2469*
–	–	–	–	–	–	*0.2*	–	–	*1.0968*	*1.2051*	*1.2129*	*1.2521*
–	–	–	–	–	–	*0.3*	–	–	*1.1015*	*1.2098*	*1.2174*	*1.2566*
–	–	–	–	–	–	*0.4*	–	–	*1.1056*	*1.2138*	*1.2214*	*1.2604*
–	–	–	–	–	–	–	*0.1*	–	*1.0878*	*1.1964*	*1.2043*	*1.2439*
–	–	–	–	–	–	–	*0.2*	–	*1.0912*	*1.1996*	*1.2075*	*1.2469*
–	–	–	–	–	–	–	*0.3*	–	*1.0945*	*1.2029*	*1.2107*	*1.2500*
–	–	–	–	–	–	–	*0.4*	–	*0.2699*	*1.2061*	*1.2138*	*1.2530*
–	–	–	–	–	–	–	–	*0.1*	*1.0214*	*1.1290*	*1.1385*	*1.1786*
–	–	–	–	–	–	–	–	*0.2*	*1.0157*	*1.1252*	*1.1357*	*1.1771*
–	–	–	–	–	–	–	–	*0.3*	*1.0098*	*1.1211*	*1.1326*	*1.1751*
–	–	–	–	–	–	–	–	*0.4*	*1.0038*	*1.1167*	*1.1292*	*1.1728*

Table 3 presented the computational results for Nusselt Numbers under varying parametric values. In Table 3, The Nu values enhanced clearly in response of higher ${\gamma}_{3}$, ${H}_{a}$, ${\gamma}_{2}$ and ${B}_{1}$ whereas it observed an opposite behavior against the rising values of ${\phi}_{1}$, ${R}_{d}$ and ${\gamma}_{4}$ in all cases. Furthermore, the Nu observed gradual slow decreasing behavior because of enhancing ${\gamma}_{1}$. The Nu result against the growing *δ * attract due to increasing scenario for the case of tetra while declined in rest of the cases.

**Table 3:** Nu variations against physical parameters

Ranges of the parameters									Nusselt number			
									Tetra	Ternary	Hybrid	Nano
${{\boldsymbol{\upphi}}}_{1}$	${\gamma}_{3}$	${\gamma}_{1}$	${H}_{a}$	${\gamma}_{2}$	${R}_{d}$	${B}_{1}$	*δ *	${\gamma}_{4}$				
*0.01*	*0.1*	*0.1*	*0.1*	*0.1*	*0.1*	*0.1*	*0.1*	*0.1*	*0.1490*	*0.1246*	*0.1161*	*0.1120*
*0.02*	–	–	–	–	–	–	–	–	*0.1484*	*0.1241*	*0.1157*	*0.1116*
*0.03*	–	–	–	–	–	–	–	–	*0.1478*	*0.1236*	*0.1152*	*0.1112*
*0.04*	–	–	–	–	–	–	–	–	*0.1400*	*0.1230*	*0.1148*	*0.1107*
–	*0.1*	–	–	–	–	–	–	–	*0.1485*	*0.1241*	*0.1157*	*0.1116*
–	*0.2*	–	–	–	–	–	–	–	*0.1490*	*0.1246*	*0.1161*	*0.1120*
–	*0.3*	–	–	–	–	–	–	–	*0.1495*	*0.1250*	*0.1165*	*0.1124*
–	*0.4*	–	–	–	–	–	–	–	*1.2088*	*0.1253*	*0.1169*	*0.1127*
–	–	*0.1*	–	–	–	–	–	–	*0.1485*	*0.1241*	*0.1157*	*0.1116*
–	–	*0.2*	–	–	–	–	–	–	*0.1485*	*0.1241*	*0.1157*	*0.1116*
–	–	*0.3*	–	–	–	–	–	–	*0.1485*	*0.1241*	*0.1157*	*0.1116*
–	–	*0.4*	–	–	–	–	–	–	*-1.2088*	*0.1240*	*0.1156*	*0.1115*
–	–	–	*0.1*	–	–	–	–	–	*0.1485*	*0.1241*	*0.1157*	*0.1116*
–	–	–	*0.2*	–	–	–	–	–	*0.1486*	*0.1242*	*0.1158*	*0.1117*
–	–	–	*0.3*	–	–	–	–	–	*0.1492*	*0.1243*	*0.1159*	*0.1118*
–	–	–	*0.4*	–	–	–	–	–	*0.1495*	*0.1244*	*0.1160*	*0.1119*
–	–	–	–	*0.1*	–	–	–	–	*0.1475*	*0.1233*	*0.1150*	*0.1109*
–	–	–	–	*0.2*	–	–	–	–	*0.1480*	*0.1237*	*0.1154*	*0.1113*
–	–	–	–	*0.3*	–	–	–	–	*0.1485*	*0.1241*	*0.1157*	*0.1116*
–	–	–	–	*0.4*	–	–	–	–	*0.1486*	*0.1245*	*0.1160*	*0.1119*
–	–	–	–	–	*0.1*	–	–	–	*0.1475*	*0.1233*	*0.1150*	*0.1109*
–	–	–	–	–	*0.2*	–	–	–	*0.1469*	*0.1228*	*0.1145*	*0.1105*
–	–	–	–	–	*0.3*	–	–	–	*0.1463*	*0.1223*	*0.1141*	*0.1101*
–	–	–	–	–	*0.4*	–	–	–	*0.1462*	*0.1219*	*0.1137*	*0.1097*
–	–	–	–	–	–	*0.1*	–	–	*0.1475*	*0.1233*	*0.1150*	*0.1109*
–	–	–	–	–	–	*0.2*	–	–	*0.2707*	*0.2269*	*0.2121*	*0.2051*
–	–	–	–	–	–	*0.3*	–	–	*0.3751*	*0.3153*	*0.2952*	*0.2861*
–	–	–	–	–	–	*0.4*	–	–	*0.4400*	*0.3915*	*0.3671*	*0.3566*
–	–	–	–	–	–	–	*0.1*	–	*0.1475*	*0.1233*	*0.1150*	*0.1109*
–	–	–	–	–	–	–	*0.2*	–	*0.1475*	*0.1233*	*0.1150*	*0.1109*
–	–	–	–	–	–	–	*0.3*	–	*0.1475*	*0.1233*	*0.1150*	*0.1109*
–	–	–	–	–	–	–	*0.4*	–	*-3.7776*	*0.1234*	*0.1151*	*0.1110*
–	–	–	–	–	–	–	–	*0.1*	*0.1490*	*0.1246*	*0.1161*	*0.1120*
–	–	–	–	–	–	–	–	*0.2*	*0.1375*	*0.1149*	*0.1071*	*0.1033*
–	–	–	–	–	–	–	–	*0.3*	*0.1277*	*0.1067*	*0.0994*	*0.0958*
–	–	–	–	–	–	–	–	*0.4*	*0.0000*	*0.0995*	*0.0927*	*0.0894*

Table 4 highlights the problem validation by comparing the current outcomes with two published studies. It is obvious that the outcomes are well aligned with them which shows the results authenticity.

**Table 4:** The validation when rest of the quantities vanish

${\gamma}_{3}$	Bachok et al. [39]	Wang et al. [40]	Current output
*0.0*	*1.232588*	*1.232588*	*1.232569*
*0.5*	*0.713295*	*0.71330*	*0.71299*
*1.0*	*0*	*0*	*0*

## Conclusions

The unsteady flow model in comparison with tetra, ternary, hybrid and simple nanofluid is inspected over a stretched Riga surface in the presence of thermal slip and convective heat condition. The consideration of concerning physical constraints depicted that:

- The convective Riga surface potentially improves the temperature with dominant thermal enhancement in tetra nanofluid than other classes. This is important for engineering applications where strong heat mechanism is required.
- The mixed convection number *δ * from 0.01 to 0.04 shows minimal contribution in the heat transfer while it has dominant role in the momentum transport.
- The unsteady ${\gamma}_{2}=\mathrm{0.1,0.3,0.5,0.7}$ and stretching ${\gamma}_{3}=\mathrm{0.2,0.4,0.6,0.8}$ strongly resists the nanofluids temperature. However, the tetra nanofluid indicates slow decline comparative to ternary, hybrid and simple nanofluids.
- The nanoparticles concentration ${\phi}_{1}=\mathrm{0.01,0.02,0.03,0.04}$ and $Rd=\mathrm{0.1,0.3,0.5,0.7}$ are examined as better heat transfer parameters while modified Hartmann number ${H}_{a}$ declines it across the Riga surface.
- The skin friction depreciates from 1.0214 to 1.0051, 1.1290 to 1.1179, 1.1385 to 1.1304 and 1.1786 to 1.1740 for tetra, ternary, hybrid and simple nanofluids while the concentration kept from 1.0% to 4.0%.
- The heat transfer gradient is examined dominant for tetra nanofluid. The computed Nusselt number is 0.1490 to 0.1400, 0.1246 to 0.1230, 0.1161 to 0.1148 and 0.1120 to 0.1107 against ${\phi}_{1}=1.0\%$ to ${\phi}_{1}=4.0\%$.

### Limitations

The presented work is limited to 2D boundary layer nanofluid flow in laminar domain across vertical Riga surface under the condition of thermal slip, radiation and transient flow. Investigation done through the use of similarities and uniform thermophysical characteristics that further connect to 3D flow with variable properties. The findings provide theoretical physical output that can be authenticated with experimental data and extended numerical models for practical applications.

### Application areas

The heat transfer using tetra nanofluid through vertical Riga surface has important applications in thermal management where electromagnetic actuation increases the boundary layer control and regulates the cooling process. The combined effects of integrated parameters have valuable role in manufacturing under high temperature, thermal collectors, and electronics cooling where the heat transfer regulation is essential. Further, the properties of tetra nanofluid coupled with Riga surface provides improved thermal progress, less energy consumption and temperature uniformity making it more applicable in industrial heat transfer applications.

### Future proposal

The potential future directions are to analyze the heat mechanism through vertically placed Riga surface for non-Newtonian and bio nanofluids under non-uniform thermophysical attributes to better characterize biomedical fluids and industrial applications. Further, the study also prolongs for energy optimization, entropy and AI schemes to obtain optimum heat mechanism and enhance the efficiency of Riga surface inspired cooling systems.

## Data Availability

The datasets used and/or analysed during the current study available from the corresponding author on reasonable request.

## Nomenclature

*u,v*: Velocities (*m/s*)
*T*: Temperature (*K*)
${\mu}_{tet}$: Dynamic viscosity (*Pa.s*)
$${\rho}_{tet}$$: Density ($$kg/{m}^{3}$$)
${k}_{tet}$: Thermal conductivity (*W/mK*)
$${\sigma}_{tet}$$: Electrical conductivity (*S/m*)
*g*: Gravitational acceleration ($$m/{s}^{2}$$)
*t*: Time (*s*)
${u}_{e}$: Free stream velocity (*m/s*)
${T}_{f}$ and ${T}_{\infty }$: Fluid and ambient temperature (*K*)
*β *: Nondimensional temperature
$$F^{\prime}$$: Nondimensional velocity
$$R{e}_{x}$$: Reynolds number
${H}_{a}$: Modified Hartmann number
*Rd*: Thermal radiation number
*Pr*: Prandtl number
*δ *: Mixed convection number
$${\gamma}_{2}$$: Unsteady parameter
$${\gamma}_{3}$$: Stretching parameter
${\gamma}_{4}$: Thermal slip parameter
$${B}_{1}$$: Biot number
$${\phi}_{1},{\phi}_{2},{\phi}_{3},{\phi}_{4}$$: Nanoparticles concentration
